# Enhancing Nasopharyngeal Carcinoma Cell Separation with Selective Fibronectin Coating and Topographical Modification on Polydimethylsiloxane Scaffold Platforms

**DOI:** 10.3390/ijms241512409

**Published:** 2023-08-03

**Authors:** M. T. Wang, S. W. Pang

**Affiliations:** Department of Electrical Engineering, Centre for Biosystems, Neuroscience, and Nanotechnology, City University of Hong Kong, Hong Kong 999077, China; mutinwang3-c@my.cityu.edu.hk

**Keywords:** nasopharyngeal epithelial (NP460) cells, nasopharyngeal carcinoma (NPC43) cells, plasma treatment, chemical coating, cell separation

## Abstract

The extracellular matrix (ECM) serves as a complex scaffold with diverse physical dimensions and surface properties influencing NPC cell migration. Polydimethylsiloxane (PDMS), a widely used biocompatible material, is hydrophobic and undesirable for cell seeding. Thus, the establishment of a biomimetic model with varied topographies and surface properties is essential for effective NPC43 cell separation from NP460 cells. This study explored how ECM surface properties influence NP460 and NPC43 cell behaviors via plasma treatments and chemical modifications to alter the platform surface. In addition to the conventional oxygen/nitrogen (O_2_/N_2_) plasma treatment, O_2_ and argon plasma treatments were utilized to modify the platform surface, which increased the hydrophilicity of the PDMS platforms, resulting in enhanced cell adhesion. (3-aminopropyl)triethoxysilane and fibronectin (FN) were used to coat the PDMS platforms uniformly and selectively. The chemical coatings significantly affected cell motility and spreading, as cells exhibited faster migration, elongated cell shapes, and larger spreading areas on FN-coated surfaces. Furthermore, narrower top layer trenches with 5 µm width and a lower concentration of 10 µg/mL FN were coated selectively on the platforms to limit NP460 cell movements and enhance NPC43 cell separation efficiency. A significantly high separation efficiency of 99.4% was achieved on the two-layer scaffold platform with 20/5 µm wide ridge/trench (R/T) as the top layer and 40/10 µm wide R/T as the bottom layer, coupling with 10 µg/mL FN selectively coated on the sidewalls of the top and bottom layers. This work demonstrated an innovative application of selective FN coating to direct cell behavior, offering a new perspective to probe into the subtleties of NPC cell separation efficiency. Moreover, this cost-effective and compact microsystem sets a new benchmark for separating cancer cells.

## 1. Introduction

Nasopharyngeal carcinoma (NPC) is a highly aggressive and metastatic cancer, and many cases are identified in southern China and Southeast Asia. Patients often do not show obvious symptoms in the early stages of the disease [1], and early diagnosis and treatment for NPC are difficult. NPC tumor is an Epstein–Barr virus-positive cancer, and it is difficult to establish the proper NPC cell lines and animal models to study this cancer. The tumor is a complex heterogeneous tissue consisting of non-cancer stromal cells and cancerous cells in the extracellular matrix (ECM). The non-cancer stromal cells co-occupy the ECM and interact with the surrounding cancer cells. Therefore, separating the cancerous NPC cells from the non-cancer stromal cells in a tumor is desirable in cancer diagnosis, monitoring, and therapy for patients with NPC. Cell separation not only is used in cancer therapy but can also benefit regenerative medicine and tissue engineering [2].

The existing cell separation techniques mainly focus on the differences in cell size, density, adherence, and antibody binding [3]. The cellular adherence technique is a simple method, and it is frequently used to isolate cells from digested or explanted primary tissues. However, false identification often occurs because of the long adherence time [3]. The density-based technique can be used to sort large numbers of cells using centrifugation. As many cell types have similar densities and sizes, centrifugation is unable to distinctly separate cells and could have a low specificity [4]. The antibody-binding method includes two techniques: fluorescence-activated cell sorting (FACS) and magnetic-activated cell sorting (MACS). The principle of these two techniques is similar, which is producing antibodies against antigens on the cell surface. The difference between these two techniques is that FACS utilizes the conjugation of fluorescent labels to antibodies and excites the bound fluorophores by laser, while MACS relies on iron oxide-containing microbeads to link the antibodies in a magnetic field. The antibody-binding method achieves high purity in isolating cells but requires high reagent and label consumption, which are costly [3,4]. Moreover, microfluidic technology has been used for the selective isolation of cells. The lateral movements of cells in various physical confinements were applied in these studies [3,5,6]. Herein, three-dimensional (3D) polydimethylsiloxane (PDMS) platforms were developed to investigate cell migration behaviors in the vertical direction to provide a low-cost and label-free method to achieve efficient cell separation.

The surface properties of the ECM have a strong influence on cell behaviors, such as adhesion, differentiation, and proliferation [6,7,8,9,10]. PDMS gained high popularity as a biocompatible material in the fabrication of devices for many biomedical applications, including the study of cellular communication, tissue engineering, and cell isolation [7,8,11,12,13]. Its unique properties provide several advantages over other biocompatible and hydrophilic polymers. Firstly, PDMS is cost effective and enables rapid prototyping, making it an attractive option for biological research. The fabrication technology for PDMS platforms and chips is straightforward and easily replicable, which contributes to its widespread use [11,12]. Secondly, PDMS exhibits low elasticity that can be conveniently adjusted by varying the ratio of the base to the curing agent. This tenability allows for a broad spectrum of material stiffness to accommodate various application requirements [14,15]. Thirdly, PDMS is gas permeable, an attribute that proves beneficial in long-term cell culture. The gas permeability facilitates the exchange of gases essential for cell survival and growth, thereby enhancing the longevity of cultured cells [16]. Lastly, the superior optical transparency of PDMS facilitates the real-time imaging and precise tracking of individual cells on the platforms, which are crucial in many fundamental studies and biochemical device applications [16,17]. Therefore, the combination of biocompatibility, cost effectiveness, ease of fabrication, tuneability, gas permeability, and optical transparency renders PDMS a preferred material for cell-based platforms in various applications. However, as PDMS is hydrophobic, it is difficult for cells to adhere to the PDMS surface [7]. Hence, the surface properties of the PDMS platforms should be modified and tailored to the intended cell studies.

Several approaches have been developed to modify the PDMS surface. Plasma treatment has been used to increase the hydrophilicity of PDMS since the process is quick and simple [18]. In plasma, the ionized gases provide reactive chemical species that change the chemical composition on the PDMS surface [18,19]. Oxygen (O_2_) and argon (Ar) plasma were shown to remove carbon on the PDMS surface and replace the methyl groups with polar groups, hence increasing the surface hydrophilicity and cell adhesion [20]. Different from plasma treatments, chemical treatments of the PDMS surface provide a stronger and more stable chemical bonding to the surface. Platforms have been coated with (3-aminopropyl)triethoxysilane (APTES) and/or fibronectin (FN) to promote cell migration. APTES, an aminosilane commonly used in the salinization process [8,21], can improve cell attachment by introducing the reactive functional group (-NH_2_) and forming covalent bonds with the cell membrane or the ECM proteins. On the other hand, APTES has been reported to act as a linker for immobilizing proteins and antibodies, hence improving cell adhesion and proliferation [7,21,22]. However, how APTES-coated PDMS surface could change cell proliferation and stabilize cell adhesion has not been thoroughly examined. Moreover, ECM proteins such as collagen, gelatin, and FN are often used to coat PDMS platforms to enhance cell attachment, proliferation, and differentiation [8,18,23]. Numerous studies have employed FN coating on platforms to study cell attachment and viability, given the diverse cell-binding properties of FN, its ability to enhance cell spreading and migration, and its role in essential biological processes [18]. FN interacts with and activates cell surface integrin receptor, which then connects with the actin cytoskeleton in cells and forms focal adhesions (FAs) to regulate cell migration [24].

The ECM functions as a complex scaffold for cells in the tissue, encompassing varied physical dimensions and surface properties that influence NPC cell migration and traversing behaviors. Previous studies have demonstrated the influential role of topographical features in the ECM in guiding cell migration, with a focus on controlling the dimensions and layouts of 3D PDMS scaffold platforms to realize the NPC cell separation [25,26,27,28,29]. The concept of utilizing these topographical features to direct cell adhesion, seeding, viability, and migration on two-dimensional surfaces has been well established [9,18,20,22,30,31,32]. Different from the previous 3D scaffold platforms, this study aimed to understand the influence of surface properties on NPC cell migration and developed a sophisticated design of the 3D scaffold platform for higher separation efficiency between nasopharyngeal carcinoma (NPC43) and epithelial (NP460) cells. This study significantly advanced our understanding of the complex interplay between chemical cues and cell behaviors, introducing a new approach to control NPC cell migration behaviors and maximize cell separation. Motivated by increasing the separation efficiency of NPC43 from NP460 cells, an aspiration unfulfilled in previous studies, novel 3D scaffold platforms with novel topographical design and biochemical modifications were developed to efficiently separate NPC43 cells from NP460 cells. By having narrow trenches with 5 µm width on the top layer of the scaffold platforms and selectively coating a low concentration FN of 10 µg/mL on the sidewalls of the top and the bottom layers, NPC43 cells were able to traverse into the 5 µm wide trenches but not the NP460 cells, resulting in a record high separation efficiency of 99.4%. In this new design, not only the physical dimension of the platforms was altered, but how the surface properties of the ECM can steer NPC cell migration was also investigated. Overall, this study presents a novel PDMS scaffold platform design with selective FN coating, contributing to a better understanding of the dynamics of cellular migration and achieving an exceptional separation efficiency of 99.4%, marking a noteworthy advancement and achieving the highest cell separation efficiency compared to previous studies. The uniqueness of this study lies in developing a microenvironment to better control NPC cell behaviors, providing a new direction for targeted interventions.

## 2. Results and Discussion

### 2.1. NP460 and NPC43 Cells on Plasma-Treated Surfaces

#### 2.1.1. Surface Energy and Roughness of Plasma-Treated Surfaces

The effects of these plasma treatments on the PDMS surface were characterized by evaluating the surface energy and the average surface roughness after exposure to the plasma. Measurement of contact angles on the PDMS layer can be used to calculate the surface energy of the PDMS. The contact angle on PDMS was measured using a contact angle meter (DropMeter A-100, Maist Vision Inspection & Measurement, Ningbo, China) by dropping the deionized water (DI) water on the PDMS. Surface energy between PDMS and DI water (σ_sl_) can be calculated based on the contact angle results and the surface energy of DI water (σ_l_). The surface energy of PDMS (σ_s_) and the surface energy between PDMS and DI water (σ_sl_) can be calculated using the following [33]:

(1) $$\sigma_{\mathrm{sl}}{=\sigma }_{l\;}{+\sigma }_{s\;}-\;2\sqrt{\sigma_{l}\sigma_{s}}e^{-{\beta (\sigma }_{l\;}-{\;\sigma }_{s}{)}^{2}}$$

(2) $$\sigma_{s}{=\sigma }_{\mathrm{sl}\;}{+\sigma }_{l}\cos\theta$$

where σ_l_ is the surface energy of DI water and is 72.8 mN/m, the coefficient β is 0.0001247, and θ is the measured contact angle.

**Figure 1:**
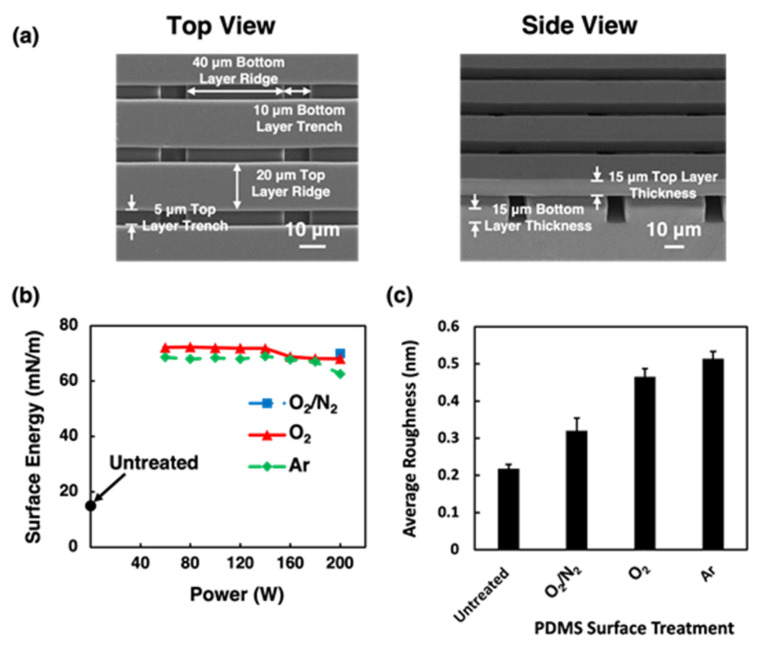
(**a**) Micrographs of top and side views of two-layer scaffold platforms with 20/5 µm wide ridge/trench (R/T) and 15 µm deep grating on top and 40/10 µm wide R/T and 15 µm deep grating on bottom. (**b**) Measured surface energy of untreated polydimethylsiloxane (PDMS) and PDMS with oxygen/nitrogen (O_2_/N_2_)-, O_2_-, and argon (Ar)- plasma treatments. (**c**) Average surface roughness of PDMS surfaces that were untreated, O_2_/N_2_ plasma at 200 W, and O_2_ and Ar plasma at 80 W.

Hydrophobic surfaces have lower surface energy, while hydrophilic surfaces have higher surface energy. Figure 1 shows the micrographs of the scaffold platforms and the surface energy variations after O_2_/N_2_, O_2_, and Ar plasma treatments. After the plasma treatments, the surface hydrophilicity of PDMS surfaces increased compared to the untreated ones with a surface energy of 15.04 mM/m. The surface energy for the plasma-treated PDMS surfaces was mostly independent of the RF power used. The mean surface energy was 70, 71, and 67 mM/m for O_2_/N_2_-, O_2_-, and Ar-treated PDMS surfaces, respectively, which was substantially higher than the untreated PDMS surfaces. The increase in surface hydrophilicity was primarily due to the removal of hydrocarbon groups and the introduction of polar functional groups [20,34]. Comparatively, O_2_/N_2_ and O_2_ plasma treatments resulted in slightly higher mean surface energy than the Ar plasma treatment. The O_2_/N_2_ plasma introduced new N- and O-containing functional groups, which were polar and hydrophilic. These groups could interact with water, thereby increasing the surface energy and hydrophilicity of the PDMS surface. Similarly, the O_2_ plasma introduced O-containing functional groups onto the PDMS surface. These groups were highly polar, thus increasing the surface hydrophilicity [18]. Contrarily, Ar is a noble gas, and Ar plasma primarily induced physical changes to the PDMS surface and removed hydrocarbon groups without introducing new functional groups. In general, Ar plasma can increase surface roughness, which, in turn, enhances hydrophilicity by increasing the surface area available [35]. In this study, the low gas flow rate and pressure applied during the Ar plasma treatment minimized the etching of the PDMS surface. Without the additional polar functional group, slightly lower surface energy was obtained for Ar plasma-treated surfaces compared to those treated with O_2_/N_2_ and O_2_ plasma. Therefore, all three plasma treatments enhanced the hydrophilicity of PDMS and made the surface suitable for cell migration characterization.

The purpose of plasma treatment is to change the chemical composition on the platform surface, and the condition was chosen so that there was no etching of the PDMS and surface smoothness had minimal changes. As shown in Figure 1c, compared to the untreated PDMS surface, the PDMS surface remained smooth after the O_2_/N_2_ plasma treatment at 200 W, with only a small increase in surface roughness from 0.22 to 0.32 nm. However, when the RF power for the O_2_ or Ar plasma was above 100 W, the PDMS surface became rough. Supplementary Figure S1a shows the average surface roughness of O_2_- and Ar-treated PDMS surfaces with RF power ranging from 60 W to 200 W. To avoid the increase in surface roughness, the RF power was kept at 80 W for O_2_ and Ar plasma treatments. The surface roughness of the O_2_ plasma-treated PDMS surface increased to 0.47 nm, and the Ar plasma-treated PDMS surface had a slightly larger surface roughness of 0.51 nm. The 3D atomic force microscopy (AFM) images of surface roughness for untreated O_2_/N_2_-, O_2_-, and Ar-treated PDMS surfaces were shown in Supplementary Figure S1b. The disparities in surface roughness were likely to be attributed to the distinct physical and chemical interactions between the plasma and the PDMS surface. The O_2_/N_2_ plasma treatment, conducted using a barrel-type plasma system, induced a lower ion energy than O_2_ and Ar plasma treatments, which were performed using a deep reactive etching (DRIE) system. The DRIE system had a rf-powered stage with a self-induced dc bias voltage, which means ions accelerated with higher ion energy towards the PDMS surface, potentially leading to higher surface roughness [36]. As the PDMS surface was bombarded with higher energy ions, this could lead to a rougher surface for O_2_ or N_2_ plasma treatment. After the plasma treatments, although the surface energy increased substantially, the treated surfaces were still very smooth with just a small increase in surface roughness [20].

#### 2.1.2. Effects of Plasma Treatments on Cell Motility

**Figure 2:**
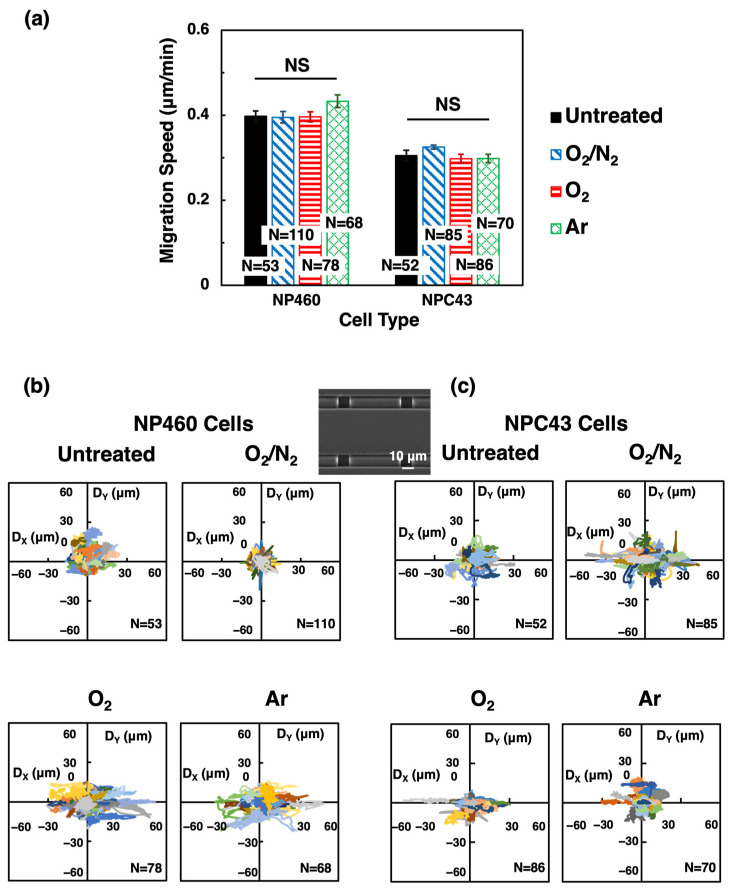
(**a**) Migration speed, (**b**,**c**) trajectories of NP460 and NPC43 cells on un-treated, O_2_/N_2_-, O_2_-, and Ar-treated two-layer scaffold platforms with 40/10 µm wide R/T on top and bottom layers. Different colors represent different cell trajectories in (**b**,**c**). One-way ANOVA with Tukey’s post hoc test; NS—not significant.

The two-layer scaffold platforms consisted of two layers of 40/10 µm wide R/T and 15 µm deep gratings, and the top layer was perpendicular to the bottom layer. The plasma systems exposed the scaffold platforms to O_2_/N_2_, O_2_, and Ar plasma to alter the surface chemistry. The increased hydrophilicity of the plasma-treated surfaces would enhance cell attachment [18]. Consistent with previous findings, NP460 cells moved faster than NPC43 cells on the scaffold platforms [25,26]. After the plasma treatments of the platforms, NP460 and NPC43 cells had similar migration speeds compared to cells on untreated platforms, as shown in Figure 2a. NP460 cells had an average migration speed of 0.41 µm/min, while NPC43 cells had an average speed of 0.31 µm/min.

The cell movements of NP460 and NPC43 cells on untreated and plasma-treated platforms over the 16 h imaging period were plotted as trajectories, as shown in Figure 2b,c. The x-axis represents alignment with the top layer grating orientation of the scaffold platform, while the y-axis corresponds to alignment with the bottom layer grating orientation. When cells were guided by the top layer grating, their trajectories would show better alignment to the x-axis. Conversely, if the cells moved randomly without any directional preference, their trajectories will not align with the x- or y-axis, as observed on the untreated platforms. When the platform was treated with O_2_/N_2_ plasma, NP460 cells continued to exhibit a random movement, with similar ranges of movement on x- and y-axes. Conversely, NPC43 cells responded differently, showing better guidance along the top layer grating orientation compared to NP460 cells after the O_2_/N_2_ plasma treatment. The O_2_ and Ar plasma treatments had a more pronounced effect on cell guidance. In Figure 2b,c, both NP460 and NPC43 cell trajectories showed a certain degree of alignment with the top layer grating orientation for O_2_ or Ar-treated platforms. It indicated that these cells were more effectively guided by the top layer gratings after the O_2_ or Ar treatments. This was evidenced by a larger number of cells that were observed to sense the edges of the top layer ridges and migrate along the trenches of the top layers. As indicated in the AFM measurements, O_2_ and Ar plasma treatments caused a larger increase in surface roughness compared to O_2_/N_2_ plasma-treated surfaces, which could provide additional topographical cues for cell guidance.

#### 2.1.3. Effects of Plasma Treatments on Cell Morphology and Traversing Behaviors

**Figure 3:**
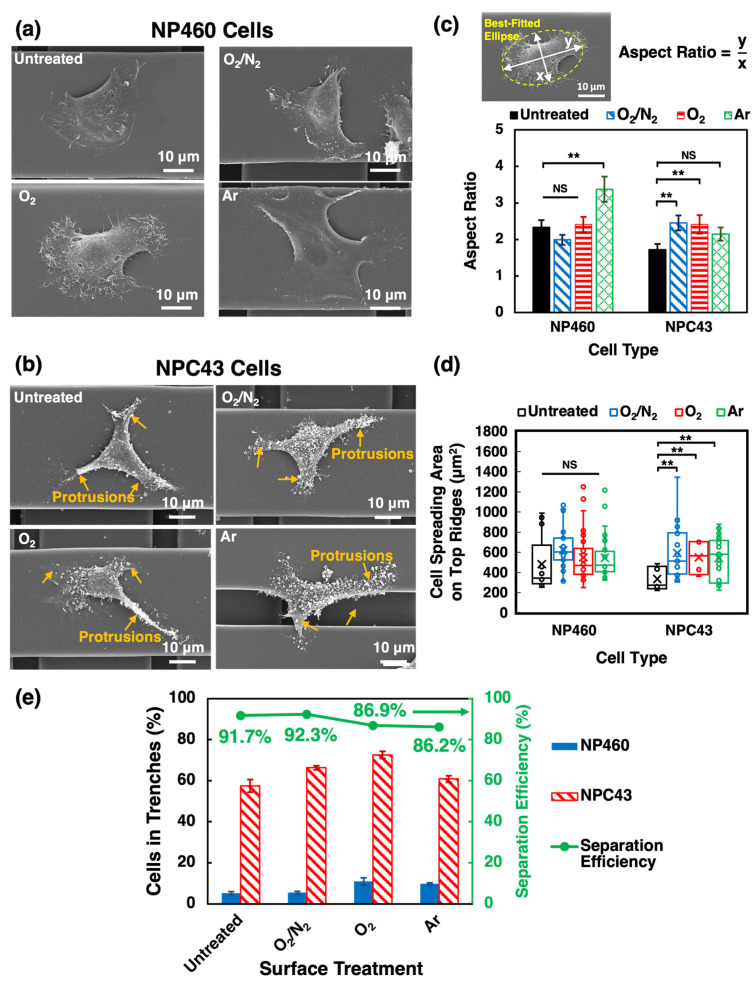
(**a**,**b**) Micrographs, (**c**) aspect ratio, (**d**) cell spreading area on top ridges, and (**e**) traversing probability and separation efficiency of NP460 and NPC43 cells on un-treated, O_2_/N_2_-, O_2_-, and Ar-treated two-layer scaffold platforms with 40/10 µm wide R/T and 15 µm deep gratings on top and bottom layers. Yellow arrows point to protrusions of NPC43 cells in (**b**). Mean values are represented with × in (**d**). One-way ANOVA with Tukey’s post hoc test; NS—not significant and ** *p* < 0.01.

The effects of different plasma treatments on NP460 and NPC43 cell morphological change are shown in Figure 3. Figure 3a,b show the morphology of NP460 and NPC43 cells on the top layer ridges of the untreated, O_2_/N_2_-, O_2_-, and Ar-treated scaffold platforms. It can be seen that NP460 cells had sheet-like lamellipodia, while NPC43 cells had long protrusions in all directions with vesicles on the cell surface [26]. To quantify the cell morphological change for cells spread on different plasma-treated platforms, the aspect ratio of NP460 and NPC43 cells was calculated, as shown in Figure 3c. Aspect ratio is defined as the ratio between the major and minor axes of a best-fitted ellipse generated via the ImageJ software based on the outline of the cell. Statistical comparisons between different groups were conducted, and one-way ANOVA with Tukey’s post hoc test was used to analyze the significant differences among various groups. Figure 3c shows that Ar-treated platforms had a substantial effect on NP460 cell elongation with an aspect ratio of 3.4 compared to 2.3 for cells on the untreated platforms. The aspect ratio of NP460 cells on the O_2_/N_2_- and O_2_-treated platforms was 2.0 and 2.4, respectively, with insignificant differences compared to cells on the untreated platforms. Cell elongation was observed for NPC43 cells on all the PDMS platforms. NPC43 cells seeded on the untreated scaffold platforms had the lowest aspect ratio of 1.7, smaller than NPC43 cells on the O_2_/N_2_- and O_2_-treated platforms with an aspect ratio of 2.5 and 2.4, respectively. NPC43 cells on the Ar-treated platforms had an aspect ratio of 2.2, still larger than cells on the untreated platforms, but the statistical difference was not significant. These observations suggested that the different plasma treatments impact cell morphology differently, probably due to varying effects on surface properties such as hydrophilicity, functional groups introduced, and surface roughness, which, in turn, influenced cell adhesion and polarization. It has been shown that higher hydrophilicity of PDMS surfaces correlated with better cell adhesion and larger cell polarization [9], hence the increased aspect ratio of NPC43 cells on plasma-treated platforms with long protrusions and filaments extending in all directions, NPC43 cells could explore and contact larger areas, potentially making them more sensitive to changes in the PDMS surface. However, Ar plasma treatment appeared to cause more significant morphological changes in NP460 cells compared to O_2_/N_2_ and O_2_ plasma treatments. This could be due to a larger increase in surface roughness induced by Ar plasma, providing additional physical cues that influence cell morphology. On the other hand, O_2_ and O_2_/N_2_ plasma treatments involved more chemical modifications to the PDMS surface due to the reactive O_2_ and N_2_-related species resulted in less drastic cell shape changes due to a more isotropic distribution of cell protrusions [35,37].

In Figure 3d, the spreading area of NP460 and NPC43 cells on the top ridges of the plasma-treated platforms was measured and compared with the untreated platforms. The spreading area of NP460 and NPC43 cells was assessed using the SEM, which provided high-resolution images of the cells. There was no statistical difference in the spreading area among NP460 cells on different platforms. The spreading area of NP460 cells on the top layer ridges was 482, 619, 548, and 545 µm^2^, respectively, on untreated, O_2_/N_2_-, O_2_-, and Ar-treated platforms. NPC43 cells had the smallest spreading area of 334 µm^2^ on the untreated top layer ridges. Substantial larger spreading areas of 593, 549, and 542 µm^2^ were obtained on O_2_/N_2_-, O_2_-, and Ar-treated surfaces. From the results shown in Figure 3c,d, all plasma-treated platforms elicited more significant responses for NPC43 cells than NP460 cells. Platforms with plasma treatments had higher surface energy, which promoted FA formation and the spreading of NPC43 cells on the surfaces [30,31,32,38]. In contrast, the epithelial NP460 cells were less sensitive to the surfaces modified by the plasma treatments compared to the carcinoma NPC43 cells [39].

The number of NP460 and NPC43 cells that migrated into the 10 µm wide trenches of the top layers during the 16 h imaging time was monitored, as shown in Figure 3e. From Figure 3e, it is seen that a majority of NP460 cells stayed on the 40 µm wide top layer ridges, and a large number of NPC43 cells migrated into the 10 µm wide trenches of the top layers on all platforms. After the two-layer scaffold platforms were treated with different plasmas, NP460 and NPC43 cell traversing behaviors were enhanced to different degrees. It has been revealed that cells in contact with various surface chemistry could modulate the FA assembly and signaling [31]. A previous study reported that plasma-treated platforms increased the ability of cells to form FA on the surfaces [29]. The traversing probability for NP460 cells was 5.2%, 5.5%, 10.9%, and 9.8% on the untreated, O_2_/N_2_-, O_2_-, and Ar-treated platforms. For NPC 43 cells, O_2_/N_2_- and O_2_-treated platform surfaces triggered more cells to move into the 10 µm wide trenches of the top layers with a traversing probability of 66.3% and 79.7%, respectively, compared to untreated platforms with a probability of 57.4%. In total, 60.9% of NPC43 cells were found in the 10 µm wide top layer trenches of the Ar-treated platforms. This may be related to the lower aspect ratio of the NPC43 cell shape on Ar-treated platforms compared to cells on O_2_/N_2_- and O_2_-plasma-treated platforms [14]. A separation efficiency of 91.7% was obtained on the untreated PDMS platforms. As an increasing number of NP460 and NPC43 cells migrated into the 10 µm wide trenches of the plasma-treated platforms, the separation efficiency was 92.3%, 86.9%, and 86.2%, respectively, on the platforms treated with O_2_/N_2_, O_2_, and Ar plasma. The above results showed that plasma treatments enhanced cell attachment, but the influence on NP460 and NPC43 cell motility was not significant. Moreover, the traversing probability of NP460 and NPC43 cells was not highly increased. Chemical modifications were designed to change the PDMS surface properties and improve cell migration and traversing behaviors.

### 2.2. NP460 and NPC43 Cells on Chemically Coated Surfaces

#### 2.2.1. Characterization of Coating on Platforms

In this study, 10% APTES or 50 μg/mL FN was coated on the scaffold platforms in several ways, including coating APTES or FN on the entire platforms, coating FN on the APTES-modified platforms, and selectively coating FN on the sidewalls of the top and bottom layers but with uncoated hydrophobic top ridges. The selective coating provided cells a higher chance to move from the top layer ridges into the top layer trenches since FN-coated sidewalls of the top layer and bottom layer attract cells to squeeze into the top layer trenches from the uncoated hydrophobic top layer ridges.

To study the FN distribution, fluorescent imaging was used to characterize the physical locations of the FN coating on the platforms, as shown in Supplementary Figure S2a–c. The FN-coated area was counterstained with Alexa Fluor 555 phalloidin, and it showed red fluorescence, while the uncoated area had a black color. The red fluorescence intensity is relative to the background intensity. It is evident that FN was coated on the top layer ridges when the platform was fully covered with the FN, as shown in Supplementary Figure S2a, with a focus on the top ridges. For APTES + FN-coated platforms, a brighter color appeared on the top layer ridges compared to FN-coated platforms with the focus on the top ridges, as shown in Supplementary Figure S2b, due to APTES enhancing additional FN coating on the platforms. In Supplementary Figure S2c, FN can only be found on the trenches of the bottom layers on the selectively coated platforms with a focus on the top ridges, as the top layer ridges were not coated with FN. The fluorescence intensity of FN- and APTES + FN-coated surfaces were measured. As APTES enables a stronger and more stable covalent attachment of FN, the fluorescence signal of FN was significantly increased on the APTES + FN-coated surface, as shown in Supplementary Figure S2d. The results show that APTES enhanced FN attachment on the PDMS surfaces, and FN could be selectively coated on the top layer trench sidewalls and bottom layer without coating the top layer ridges.

#### 2.2.2. Effects of Chemical Coatings on Cell Motility

**Figure 4:**
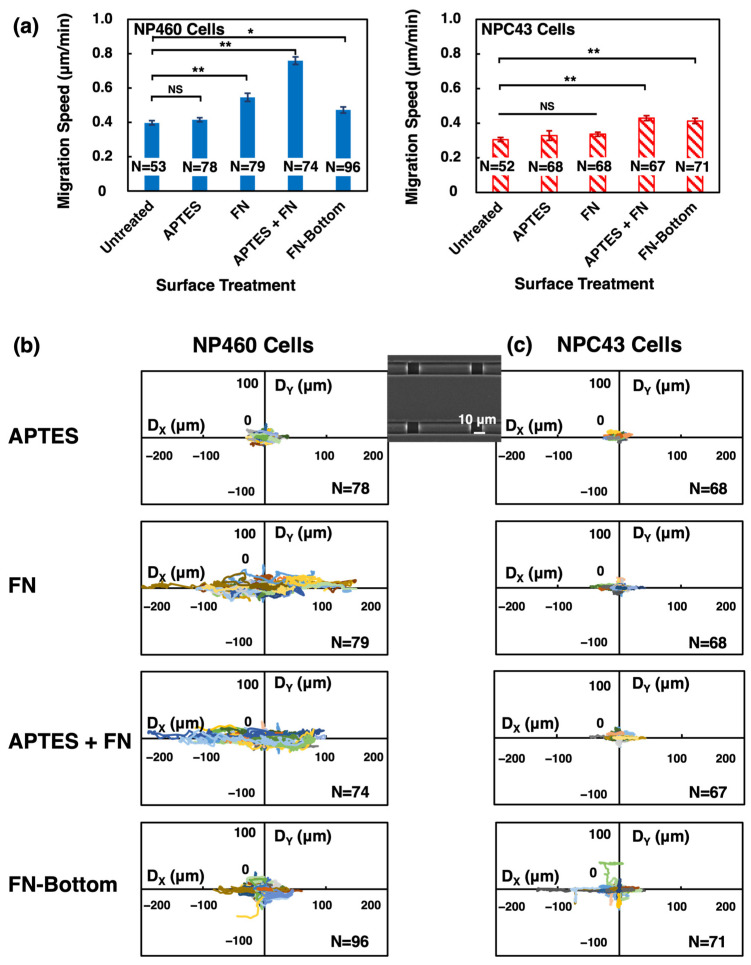
(**a**) Migration speed and (**b**,**c**) trajectories of NP460 and NPC43 cells on two-layer scaffold platforms with 40/10 µm wide R/T and 15 µm deep gratings on top and bottom layers completely coated with (3-aminopropyl)triethoxysilane (APTES), fibronectin (FN), APTES + FN, and FN coated only on top layer sidewalls and bottom layer. One-way ANOVA with Tukey’s post hoc test; NS—not significant, * *p* < 0.05, and ** *p* < 0.01.

As the chemically modified PDMS surfaces considerably increased the hydrophilicity of PDMS [40], cell motility was accordingly influenced and reflected in the cell migration speed and trajectories, as shown in Figure 4. NP460 cells on the APTES-coated platforms had a similar migration speed as on the untreated platforms of 0.42 µm/min. On FN-, APTES +FN-, and FN selectively coated on the top layer sidewalls and bottom layer platforms, faster cell migration speeds of 0.55, 0.76, and 0.47 µm/min, respectively, were measured, as shown in Figure 4a. These results suggest that endothelial cell adhesion, spreading, and proliferation could be significantly increased with protein coatings. The interactions between the cell surface and FN coating on the platform surface initiated a series of intracellular signaling pathways that affect cell migration [18]. Moreover, APTES enhanced FN coating on the PDMS platforms. Consequently, a substantial increase in migration speed appeared on the APTES + FN-coated PDMS platforms compared to FN-functionalized surfaces. NP460 cells had a slower migration speed on the selectively coated platforms without FN coating on the top layer ridges, resulting in the hydrophobic surface on the ridges. However, the movements of NP460 cells became faster once they migrated from the untreated, hydrophobic top layer ridges to the FN-coated trenches of the top layers.

As shown in Figure 4a, the migration speed of NPC43 cells on APTES- and FN-coated platforms was 0.33 and 0.34 µm/min, respectively, which was similar to the migration speed of 0.31 µm/min for cells migrated on the untreated platforms. When APTES or FN was coated on the entire platforms, some NPC43 cells were partially traversed into the 10 µm wide top layer trenches through the long protrusions, while parts of the cells were still attached to the top ridges. These partially traversed NPC43 cells could not migrate freely on the platforms, resulting in a low migration speed. Higher migration speeds of 0.43 and 0.41 µm/min were obtained when NPC43 cells migrated on the APTES + FN- and selectively FN-coated platforms, respectively. Consistent with NP460 cells, NPC43 cells had the highest migration speed on the APTES + FN-coated platforms due to APTES promoting additional FN binding to the PDMS platforms. With FN selectively coated on the sidewalls of the top layer and bottom layer of the scaffold platforms, more NPC43 cells were attracted to move into the 10 µm wide top layer trenches, and these cells migrated faster in the narrow trenches compared to moving on the top layer ridges [41].

The trajectories of NP460 and NPC43 cells on chemically coated platforms were plotted, as shown in Figure 4b,c. NP460 and NPC43 cells migrated randomly and were not guided by the top layer grating on APTES-coated platforms. On the contrary, the range of NP460 cell movement increased on the FN- and APTES + FN-coated platforms, and they showed strong guidance along the top layer grating with faster migration speed, as shown in Figure 4b. When FN was coated selectively on the sidewalls of the top layer trenches and the bottom layer, NP460 cells had random movements on the untreated, hydrophobic top layer ridges, while fully traversed NP460 cells were guided in the 10 µm wide top layer trenches. In Figure 4c, NPC43 cells were guided along the top layer grating as more NPC43 cells moved into the 10 µm wide top layer trenches on the FN- and APTES + FN-coated platforms. Some NPC43 cells moved back and forth between the ridges and trenches of the top layer. Therefore, the range of NPC43 cell trajectories on the FN- and APTES + FN-coated platforms was small. On platforms with FN coating on the sidewalls of the top and bottom layers, some NPC43 cells migrated into the trenches of the bottom layer, resulting in trajectories along the x-axis of the top layer grating and along the y-axis of the bottom layer trenches.

#### 2.2.3. Effects of Chemical Coatings on Cell Morphology and Traversing Behavior

**Figure 5:**
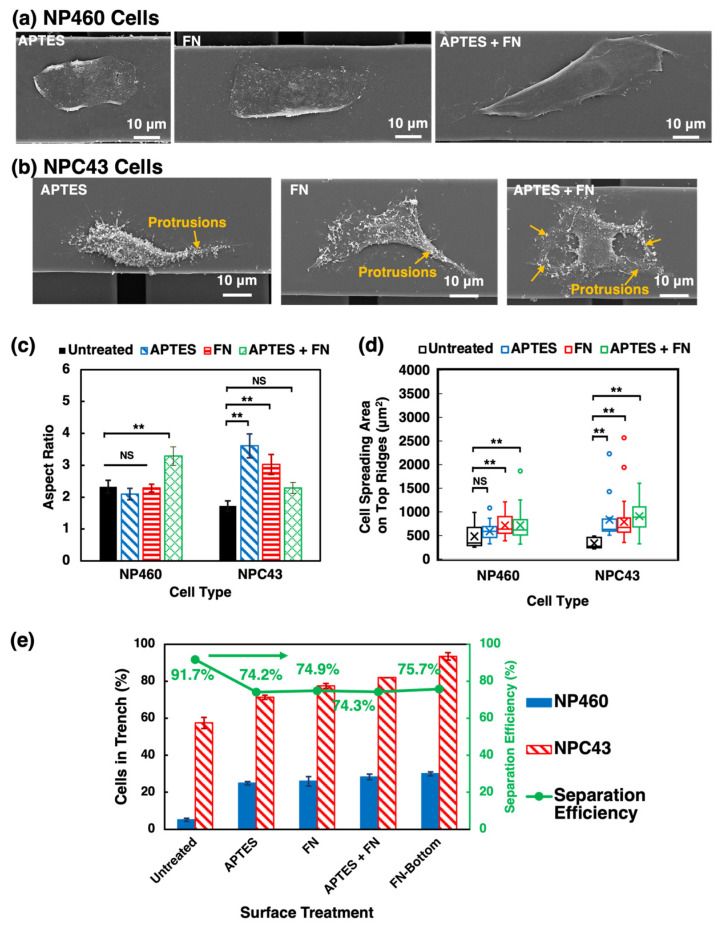
(**a**,**b**) Micrographs, (**c**) aspect ratio, (**d**) cell spreading area on top ridges, and (**e**) traversing probability and separation efficiency of NP460 and NPC43 cells on two-layer scaffold platforms with 40/10 µm wide R/T and 15 µm deep gratings on top and bottom layers completely coated with APTES, FN, APTES + FN, and FN coated only on top layer sidewalls and bottom layer. Yellow arrows point to protrusions of NPC43 cells in (**b**). Mean values are represented with × in (**d**). One-way ANOVA with Tukey’s post hoc test; NS—not significant and ** *p* < 0.01.

The above results show that NP460 and NPC43 cells behaved differently when they were on platforms with different treatments. Figure 3a,b and Figure 5a,b show the cell morphology of NP460 and NPC43 cells on the 40 µm wide ridges of the top layers of untreated and chemically coated platforms. Compared with the cells on the untreated platforms, NP460 cells on the three chemically coated platforms displayed spread-out sheet-like lamellipodia on the top layers. On the other hand, NPC43 cells have long protrusions from their cell bodies when attached to the top layer ridges. In particular, the morphological changes were apparent when NP460 and NPC43 cells were cultured on the APTES + FN-coated platforms. This could be related to the surface functionalization, as APTES enhanced FN binding on the platform surface, as confirmed by the enhanced FN fluorescence signal shown in Supplementary Figure S2. To compare and quantify the morphological differences of NP460 and NPC43 cells on platforms with various coatings, the aspect ratio and spreading area were calculated, as shown in Figure 5c,d. NP460 cells on the APTES + FN-modified surfaces displayed an elongated cell shape with a high aspect ratio of 3.3. Whereas the aspect ratio of NP460 cells on the untreated, APTES-, and FN-coated platforms was similar, with a lower aspect ratio of 2.2. Figure 5d shows that the spreading area of NP460 cells on the 40 µm wide ridges of the top layers increased on platforms treated with FN or APTES + FN compared to the untreated or APTES-coated platforms. APTES pre-coated PDMS surfaces promoted more FN binding on PDMS, which made NP460 cells migrate faster with a larger cell aspect ratio and spreading area [7,42]. NPC43 cells migrated on the APTES-, FN-, and APTES + FN-modified platforms had increased aspect ratios of 3.6, 3.0, and 2.3, respectively, compared to 1.7 on the untreated platforms. In addition, NPC43 cell spreading area increased on all chemically coated platforms, especially on the APTES + FN-coated platforms with a mean spreading area of 843 µm^2^, compared to 334 µm^2^ on the untreated platforms. Such phenomenon of increased cell area was also reported in the previous study, showing how FN coating affected prostate cancer cell morphology [43]. Although the elongation of NPC43 cells was not as much on the APTES + FN-coated platforms compared to cells on the APTES- and FN-coated platforms, NPC43 cells had elongated protrusions that spread over larger areas. In general, different chemical coatings on the PDMS surface induced an increase in the cell aspect ratio and spreading area compared to the untreated platforms.

As shown in Figure 5e, the ability of NP460 and NPC43 cells to squeeze into the 10 µm wide trenches of the top layers was boosted with chemical coatings on the platforms. Although the effects of APTES on NP460 cell motility and morphological change were not significant, more NP460 cells on the APTES-modified platforms were able to migrate into the 10 µm wide trenches of the top layer with a traversing probability of 24.9%. It might be related to the increased hydrophilicity of the platforms, which enhanced cell adhesion and migration [8]. On the other hand, 71.3% of NPC43 cells were found in the top layer trenches during the 16 h imaging time, most likely due to their elongated cell shape and extension of the long protrusions. On FN- and APTES + FN-coated platforms, although more cells could migrate into the 10 µm wide trenches, some NP460 and NPC43 cells also migrated from the trenches back to the top ridges and moved back and forth between the ridges and trenches. The highest traversing probability for NP460 and NPC43 cells was obtained on the selectively coated platforms with FN coated on the top layer trench sidewalls and the bottom layer. Both NP460 and NPC43 cells had a higher traversing probability of 30.0% and 91.9%, respectively. The FN coating on the trench sidewalls of the top and bottom layers attracted more cells into the narrow trenches by promoting the assembly of FAs, which play a role in force transmission and regulate migration [24]. The hydrophobic ridges of the top layers hindered the cells from returning to the top layer ridges. Additionally, FN coating on the bottom layer allowed a few NPC43 cells to traverse to the bottom layers. As a result of the chemical coatings, the traversing probability of NP460 cells increased from 5.2% on the untreated platforms to over 25% on the chemically coated platforms, whereas the traversing probability of NPC43 cells increased from 57.4% to over 70%. Taken together, both NP460 and NPC 43 cells traversing probability were highly increased on all chemically modified platforms, but the separation efficiency of NPC43 cells decreased compared to the untreated platforms. The separation efficiency was defined as the ratio between the traversing probability of NPC43 cells and the sum of the traversing probability of NP460 and NPC43 cells. Therefore, as the traversing probability of NP460 cells increased, the separation efficiency decreased. Accordingly, the separation efficiency of NPC43 cells decreased from 91.7% on the untreated platforms to 74.2%, 74.9%, 74.3%, and 75.7%, respectively, on APTES-, FN, APTES + FN-, and selective FN-coated platforms. To efficiently separate NPC43 cells from NP460 cells, a design combining the physical layout and chemical modification was applied to the selectively FN-coated platforms.

### 2.3. Separation of NP460 Cells from NPC43 Cells by Platform Design and Chemical Coating

This study aims to develop a novel design of 3D scaffold platforms for maximizing the separation efficiency of NPC43 cells from NP460 cells, an aspiration unfulfilled in previous studies. From the results shown above, platforms with different plasma treatments and chemical coatings could effectively increase the surface hydrophilicity, hence promoting better cell adhesion and faster migration. Our results showed that selective FN-coated platforms could attract more cells to migrate into the trenches of the top layers, while cells rarely returned to the untreated top layer ridges as the top surface was hydrophobic. As shown in the Supplementary video S1a, with FN selectively coated on the top layer sidewalls and bottom layers, more NP460 and NPC43 cells were able to migrate into the 10 µm wide trenches by stretching their lamellipodia or long protrusions to contact the sidewalls of the trenches. Some cells could further move to the bottom layer since the bottom layer was also coated with FN, which was seldom observed in our previous studies [5,14,25,26]. However, the selective coating of the platforms enhanced both NP460 and NPC43 cell traversing capabilities. To increase the separation efficiency of NPC43 cells from NP460 cells on the selectively coated platforms, two approaches were investigated to hinder NP460 cells from moving into the trenches of the top layers. The first approach was to use narrower trenches of 5 μm width on the top layer of the scaffold platforms. With single-layer platforms, nearly no NP460 or NPC43 cells could migrate into the 10 μm wide trenches [25]. Only when two-layer scaffold platforms were used with the presence of the bottom layers, many more NPC43 cells could move into the 10 or 5 µm wide trenches of the top layers, as the sidewalls of the bottom layer trenches provided additional contact areas for cells to attach. The narrower 5 μm wide trenches would limit most of the NP460 cells to migrate into the trenches while still allowing many NPC43 cells to squeeze through, especially when the sidewalls of top layer trenches and bottom layers were coated with 50 μg/mL FN. Typical NP460 and NPC43 cell sizes are 15–20 μm, but NPC43 cells are more flexible and deformable, which allows them to squeeze into the narrower trenches [14,25,28]. The second approach was to decrease the concentration of the FN coating on the sidewalls of the top layer trenches and the bottom layer from 50 μg/mL to 10μg/mL to lower the attraction force for NP460 cells to move into the 5 μm wide trenches. Meanwhile, as NPC43 cells are more deformable, many NPC43 cells could still squeeze into the 5 μm wide trenches, hence increasing the cell separation efficiency of NPC43 cells from NP460 cells.

**Figure 6:**
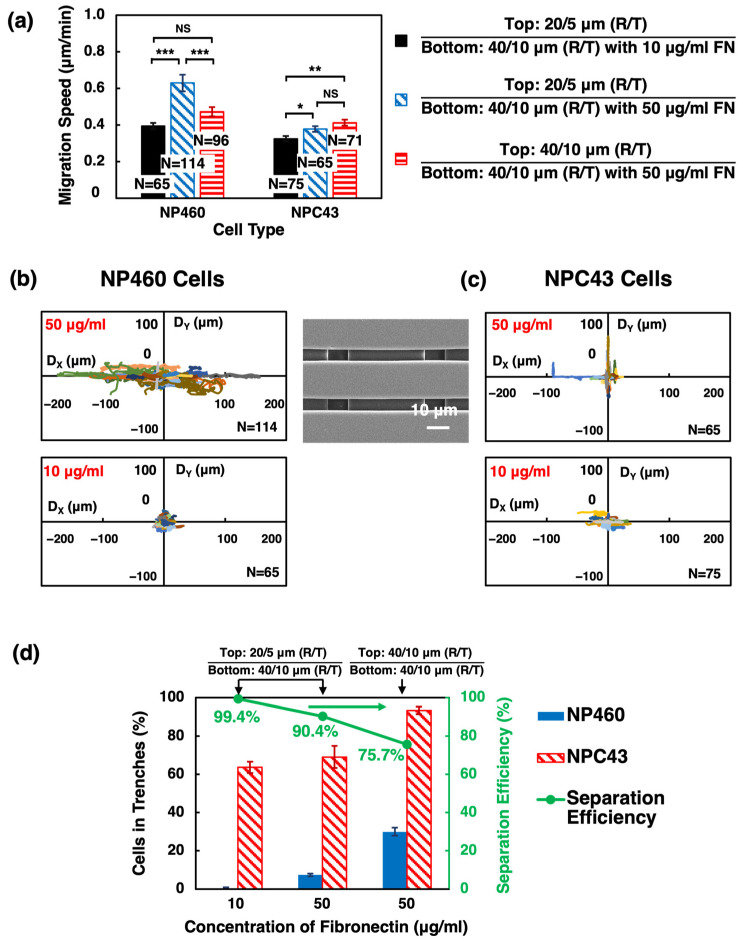
(**a**) Migration speed, (**b**,**c**) trajectories, and (**d**) traversing probability and separation efficiency of NP460 and NPC43 cells on two-layer scaffold platforms with 20/5 or 40/10 µm wide R/T on top layer and 40/10 µm wide R/T on bottom layer. Platforms were coated with 10 or 50 µg/mL FN on top layer sidewalls and bottom layer. One-way ANOVA with Tukey’s post hoc test; NS—not significant, * *p* < 0.05, ** *p* < 0.01, and *** *p* < 0.001.

For the first approach, 5 µm wide trenches on the top layer were developed. Platforms were selectively coated with 50 µg/mL FN, and the top layer of the scaffold platforms was designed to be 20/5 µm wide R/T, while the bottom layer was still 40/10 µm wide R/T. As shown in Figure 6a–c, with 5 µm wide trenches on the top layer, NP460 cells were guided along the top layer grating orientation on the 20/5 µm wide R/T top layer with a faster migration speed of 0.63 µm/min compared to platforms with 40/10 µm wide R/T on the top layer. Better guidance and faster speed were observed for NP460 cells that stayed on the top layer ridges and moved along with the ridges. In contrast, the migration speed of NPC43 cells was not affected by the topographical change in the top layer with a slower migration speed of 0.38 µm/min, similar to cells on the scaffold platforms with 40/10 µm wide R/T on the top layer. While many NPC43 cells could move into the 5 µm wide trenches of the top layer, even more, NPC43 cells could move into the 10 µm wide trenches of the bottom layers, as shown in Figure 6c. In Supplementary Video S1b, it is evident that NP460 cells took a longer time to deform their cell bodies to fit into the 5 µm wide trenches. Even after they were in the 5 µm wide trenches, NP460 cell movements were restricted. On the other hand, NPC43 cells could move along the 5 µm wide trenches of the top layer, and many could migrate to the bottom layer. The separation efficiency was defined as the ratio between the traversing probability of NPC43 cells and the sum of the traversing probability of NP460 and NPC43 cells. Narrower trenches could substantially hinder the traversing behaviors of NP460 cells. With 50 μg/mL FN coating on platforms, most NP460 cells migrated along or across the 20 µm wide ridges on the top layer, and few NP460 cells could squeeze into the 5 µm wide trenches. Therefore, the traversing probability of NP460 cells decreased significantly from 30.0% for 10 µm wide trenches to 7.3% for 5 µm wide trenches. Meanwhile, 69.1% of NPC43 cells could migrate into the 5 µm wide trenches. Accordingly, a higher separation efficiency of 90.4% was achieved on the selectively coated two-layer scaffold platforms with 5 µm wide trenches on the top layers, as shown in Figure 6d.

Apart from designing narrower trenches on the top layer to hinder NP460 cell traversing capability, a lower concentration of FN was also used as the second approach to further decrease the ability of NP460 cells to squeeze into the 5 µm wide trenches of the top layer. As shown in Figure 6a, the migration speed and trajectories of NP460 and NPC43 cells were investigated. When the concentration of FN was decreased from 50 to 10 µg/mL, NP460 and NPC43 cells had lower migration speeds of 0.39 and 0.33 µm/min, respectively. In addition, the ranges of NP460 and NPC43 cell migration trajectories were also reduced, as shown in Figure 6b,c. With 10 µg/mL FN coated selectively on the platforms, NP460 cells displayed no guidance from the grating, and NPC43 cells showed limited guidance. The reduced FN concentration used for the selective coating resulted in almost no NP460 cells in the 5 µm wide trenches of the top layer, and an extremely low traversing probability of 0.4%, The traversing probability for NPC43 cells was reduced to 63.6%, as shown in Figure 6d. Therefore, the separation efficiency of NPC43 cells increased to 99.4%, as the traversing probability of NP460 cells was greatly hindered, while many NPC43 cells could still move into the 5 µm wide trenches of the top layer with 10 µg/mL FN coated selectively on the top layer sidewalls and bottom layer.

## 3. Materials and Methods

### 3.1. Fabrication Technology for Biomimetic Two-Layer Scaffold Platforms

The fabrication details for the biomimetic scaffold platforms were described previously [5,25,26]. Briefly, grating patterns were transferred onto a silicon (Si) wafer via photolithography and dry etching and coated with a thin anti-sticking hydrophobic layer of trichloro(1H, 1H, 2H, 2H-perfluorooctyl)silane (FOTS) to form the Si stamp. By casting liquid PDMS, which was prepared by mixing the prepolymer with the curing agent (Dow Corning Sylgard 184 Kit, Dow Chemical, Cheadle, UK) at a ratio of 10:1, on the Si stamp and then baked on the hot plate at 80 °C for 6 h, it formed the bottom layer of the platform consisting of 40/10 μm wide ridge/trench (R/T) and 15 μm deep grating. This PDMS layer was peeled from the Si stamp. The top layer of the platform was generated using the reversal imprint lithography (EITRE^®^ 6, Obducat, Lund, Sweden). The top layer PDMS grating was transferred from a Si stamp coated with FOTS onto the bottom PDMS grating on the Si substrate coated with a mixture of 3-methacryloxypropyltrichlorosilane and FOTS in a 4:1 ratio. These top and bottom PDMS layers were exposed to an oxygen/nitrogen (O_2_/N_2_) plasma with 400/400 sccm O_2_/N_2_, 150 mTorr pressure, and 200 W RF power for 75 s, which promoted the adhesion between these two layers when they were stacked together. Figure 1a shows the structure of a two-layer scaffold platform with 20/5 µm wide R/T and 15 µm deep grating on top and 40/10 µm wide R/T and 15 µm deep grating on the bottom. The two layers of gratings were oriented with a 90° offset and perpendicular to one another.

### 3.2. Surface Modification of PDMS Platforms

PDMS elastomers have been extensively used as biocompatible materials in many biological applications due to their advantageous properties. However, the hydrophobic nature of the PDMS surface presents a drawback for cell attachment [7]. To overcome this issue, plasma treatments, known for their efficacy in modifying surface properties using ionized gases, were applied in this study [44]. Specifically, O_2_/N_2_, O_2_, and Ar plasma treatments were designed to enhance the hydrophilicity and alter the chemical composition on the PDMS surface. The prepared platforms were mounted on Si wafers and exposed to the plasma. A gentle approach using a barrel plasma system (GIGAbatch 310M, PVA TePla, Wettenberg, Germany) was employed to generate low-energy reactive species, a common technique to make the PDMS surface hydrophilic for cell studies [5,25,26,27,28]. In addition, O_2_ and Ar plasma treatments were carried out using a different plasma system (LPX ICP-SR, SPTS, Newport, UK) and two separate RF power supplies. These treatments aimed to introduce low-energy reactive species on the PDMS surface. The conditions for O_2_ and Ar plasma treatments were 50 sccm gas flow rate, 50 mTorr pressure, and RF power ranging from 60 to 200 W for 60 s. The varying platen power was strategically designed to study the relationship between increasing power and surface hydrophilicity. Upon plasma treatment, all platforms were preserved in DI water to sustain the increased hydrophilicity of the PDMS surface, thus preparing them for subsequent cell studies [45].

Other than plasma surface treatments, chemical coatings were also applied. The PDMS platforms were chemically coated under four different conditions, namely APTES, FN, APTES + FN, and FN-bottom coatings. To ensure uniform coating of the scaffold platforms, the platforms were treated with an O_2_/N_2_ plasma as described above for 75 s before coating. For APTES and APTE + FN coated PDMS platforms, they were immersed in APTES (10% in ethanol, Sigma-Aldrich, St. Louis, MO, USA) at 50 °C for 2 h. The platforms were then washed three times in DI water. For platforms with APTES + FN coatings, the platforms were coated with FN (50 μg/mL in DI water, Cytoskeleton, Denver, CO, USA) after the APTES coating and stored at 4 °C for 16 h. Likewise, PDMS platforms with FN coating were coated with FN and stored at 4 °C for 16 h. For FN-bottom coating, FN was coated only on the top layer sidewalls and the bottom layer by injecting the FN solution via the inlet on the bottom layer at 4 °C for 16 h while a flat PDMS plate covered the top ridges, as shown in Supplementary Figure S3. After the chemical treatments, the coated PDMS platforms were kept in the phosphate saline buffer (PBS, Life Technologies, Carlsbad, CA, USA).

### 3.3. Nasopharyngeal Epithelial and Carcinoma Cell Culture 

Nasopharyngeal NP460 and NPC43 cells [46] were incubated at 37 °C in a 5% CO_2_ incubator. NP460 cells were maintained in the medium using a 1:1 mixture of EpiLife^TM^ medium with 1% EpiLife^®^ defined growth supplement (Life Technologies, Carlsbad, CA, USA) and defined keratinocyte serum-free medium with 0.2% defined keratinocyte growth supplement (Life Technologies, Carlsbad, CA, USA). Then, 1% penicillin-streptomycin-glutamine (PSG, Life Technologies, Carlsbad, CA, USA) was added to the NP460 cell culture medium. NPC43 cells were cultured in Roswell Park Memorial Institute 1640 medium (Life Technologies, Carlsbad, CA, USA) supplemented with 10% fetal bovine serum (FBS, Life Technologies, Carlsbad, CA, USA), 1% PSG, and 0.2% 2 mM rock inhibitor Y-27632 (Enzo Life Sciences, Farmingdale, NY, USA). The NP460 and NPC43 cell culture media were replaced every two days, and the cells were passaged every three days or sooner when they reached 80% confluence. Cells were discarded after 20 passages.

### 3.4. Fluorescent Imaging of Fibronectin 

After FN coating, the platforms were immersed in the primary antibody mouse anti-vinculin (1:200, Sigma-Aldrich, St. Louis, MO, USA) diluted in PBS at 4 °C overnight to immobilize the stain for fluorescent imaging. After washing with 1 × PBS three times and for 10 min each time, the platforms were incubated in dark for 2 h with Alexa Fluor 555 phalloidin (1:250, Sigma-Aldrich, St. Louis, MO, USA) diluted in PBS to stain the FN. A confocal microscope (SP8, Leica, Wetzlar, Germany) was then used to capture the fluorescent images of FN in platforms, showing the physical locations covered by FN.

### 3.5. Time-Lapse Imaging and Data Analysis

A 35 mm diameter glass-bottom confocal dish (101350, SPL Life Science, Pocheon-si, Republic of Korea) was cleaned and turned hydrophilic by treating it with an O_2_/N_2_ plasma at flow rates of 400/400 sccm O_2_/N_2_, 150 mTorr pressure, and 200 W RF power for 75 s. The PDMS scaffold platforms were then attached to the dish. The cell seeding density of NP460 and NPC43 cells on the platforms was controlled to be 9 × 10^4^ cells/mL with even distribution across the platforms. After the 6 h initial cell attachment to the platforms, the culture medium was renewed with a mixture of the culture medium and CO_2_-independent medium (Life Technologies, USA) with 10% FBS, 1% PSG, and 1% GlutaMAX supplement (Life Technologies, USA) in a 1:1 ratio. Cells were maintained at 37 °C for the duration of the imaging time. Cell migration on the platforms was monitored every 5 min for 16 h using a Nikon upright microscope (Eclipse Ni-E, Nikon, Duesseldorf, Germany) with a 20× objective.

NP460 and NPC43 cell movements were tracked using the NIH ImageJ software (version 1.52p) with the manual tracking plugin. Only single live cells that did not divide or interact with other cells were tracked. Although the number of tracked cells varied in each run, each experimental condition was repeated three times to ensure a sufficient number (N ≥ 50) of cells for analysis. Cell migration speed and trajectory were calculated, and the total number of analyzed cells (N) from all repeated experiments was labeled in the charts. The cell shape was outlined to measure the cell projected area and aspect ratio. The aspect ratio was defined as the major axis divided by the minor axis of a fitted ellipse. The percentage of traversing cells was defined as the number of traversed cells staying in the 5 or 10 µm wide trenches of the top layers for at least 10 h during the 16 h imaging time among the total number of tracked cells. Surface energy and roughness analysis were used to characterize the plasma-treated surface. The surface roughness of the plasma-treated PDMS was quantitatively assessed using an atomic force microscope (AFM5100N, Hitachi, Chiyoda City, Japan) in tapping mode. This technique employed a scanning cantilever with a spring constant of 40 N/m. For each sample, images were captured from three different positions to ensure representative measurements. The scanning was conducted at a frequency of 0.86 Hz, and the resolution of the captured images was set at 512 pixels. The average surface roughness was subsequently computed using the AFM5000 Ⅱ software (version 7.03J2). To test the statistical significance of the results, one-way analysis of variance (ANOVA) with Tukey’s post hoc test was performed to compare the mean values of two or more groups. All data were shown as mean ± standard error of the mean.

Cell separation was defined as the ratio between the traversing probability of NPC43 cells and the sum of the traversing probability of NP460 and NPC43 cells as shown below in the following formula:

(3) $$\mathrm{Separation}\;\mathrm{Efficiency}=\frac{\mathrm{Traversing}\;\mathrm{Probability}\;\mathrm{of}\;\mathrm{NPC}43\;\mathrm{Cells}}{\mathrm{Traversing}\;\mathrm{Probability}\;\mathrm{of}\;\mathrm{NP}460+\mathrm{NPC}43\;\mathrm{Cells}}$$

### 3.6. Scanning Electron Microscopy 

After NP460 and NPC43 cells were seeded on the treated platforms for 22 h, the cells were washed with 1 × PBS two times and then fixed with 4% paraformaldehyde (Sigma-Aldrich, USA) for 20 min at 22 °C. Dehydration of cells was then performed via immersion in DI water and a series of ethanol (30%, 50%, 70%, 80, 90%, 95%, and 100%) for 5 min for each concentration. The samples were dried using the critical point dryer (EM CPD300, Leica, Germany) for 4 h and coated with a thin layer of gold before being captured using a scanning electron microscope (SEM, Schottky Field Emission SU5000, Hitachi, Japan).

## 4. Conclusions

In this study, PDMS scaffold platforms were fabricated using imprint technology for separating NPC43 cells from NP460 cells with specific physical dimensions and selective chemical coating. To address the hydrophobic nature of PDMS, various plasma treatments and chemical coatings were applied to modify the platform surface properties. The influence of different surface modifications on cell motility and morphology was analyzed. While plasma treatments significantly enhanced the hydrophilicity of the PDMS platform surface and facilitated cell attachment, they did not produce significant changes in NP460 and NPC43 cell migration or traversing behaviors. In contrast, chemical coatings proved to be more effective in altering the cell migration behaviors compared to plasma treatments, leading to increased cell migration speed and a wider range of migration trajectories. Selective FN coating on the platforms attracted a large number of NP460 and NPC43 cells into the 10 μm wide top layer trenches. By incorporating narrower trenches with 5 µm width on the top layer and a low concentration of 10 µg/mL FN selectively coated on the sidewalls of the top and bottom layers, a remarkable separation efficiency of 99.4% was achieved, enabling the effective screening of NPC43 cells from NP460 cells.

Overall, this work demonstrated the efficient separation of NPC43 cells from NP460 cells using narrow trenches and selective FN coating on 3D scaffold platforms. Imprint lithography was utilized for platform fabrication, and this technology facilitated the precise creation of the desired topographical features. Selective FN coating of the PDMS platforms was applied by injecting FN solution into the two-layer scaffold platforms, which coated all the surfaces of the scaffolds, including the sidewalls, except the ridges of the top layer as the top surface was covered by a PDMS sheet. Functioning as a filter, the two overlaid gratings on the scaffold platforms allowed only NPC43 cells to traverse through the 5 µm wide trenches of the top layers, while NP460 cells stayed on the top layer ridges. This selective traversing ability was attributed to the presence of FN coating on the trench sidewalls, which exerted an attractive force for the NPC43 cells, promoting their migration into the narrow layer. Conversely, the hydrophobic nature of the top layer ridges acted as a barrier, preventing cells from moving back to the upper surface.

What sets this method apart from other cell separation techniques is its utilization of natural cell-ECM interactions, while other techniques rely on cell size, fluorescent biomarkers, and magnetic markers to separate cells. In addition, a major advantage of this platform design is its capability for the real-time observation of cell migration speed, trajectory, and morphology. This can be achieved without the need for specific biomarkers or labeling, which simplifies the separation process without additional chemicals and also provides valuable insight into the dynamic nature of cell migration. The findings of this study hold great promise for the field of tissue engineering and offer potential applications in early disease diagnoses and therapeutic interventions. The development of these PDMS scaffold platforms, combining topographical and selective chemical modifications, opens up possibilities for creating a valuable microsystem that can advance tissue engineering and facilitate targeted interventions in the diagnosis and treatment of diseases. This innovation signifies a major advancement in cell separation techniques, heralding a new era of efficiency and precision in various biomedical applications.

## Data Availability

All data generated or analyzed during this study are included in this published article and its Supplementary Materials file.

## Supplementary Materials

The supporting information can be downloaded at: https://www.mdpi.com/article/10.3390/ijms241512409/s1.
