# Supplementary material for: Enhancing Nasopharyngeal Carcinoma Cell Separation with Selective Fibronectin Coating and Topographical Modification on Polydimethylsiloxane Scaffold Platforms
Source: Int J Mol Sci. 2023 Aug 3;24(15):12409. doi: 10.3390/ijms241512409 (PMC10418797; doi:10.3390/ijms241512409)
Supplement: Supplementary file 1 [file ijms-24-12409-s001.zip › Cell Separation-Supplementary Materials-Proof.pdf]

Supplementary Information for:

Enhancing Nasopharyngeal Carcinoma Cell Separation with Selective Fibronectin Coating and Topographical Modification on Polydimethylsiloxane Scaffold Platforms

M. T. Wang and S. W. Pang*
Department of Electrical Engineering
Centre for Biosystems, Neuroscience, and Nanotechnology
City University of Hong Kong, Hong Kong, 999077, China

*Corresponding Author:
S. W. Pang (pang@cityu.edu.hk)
Department of Electrical Engineering
City University of Hong Kong
83 Tat Chee Avenue, Kowloon
Hong Kong, China
Phone: +852 3442 9853
Fax: +852 3442 0562

Supplementary Figures and Supplementary Figure Legends

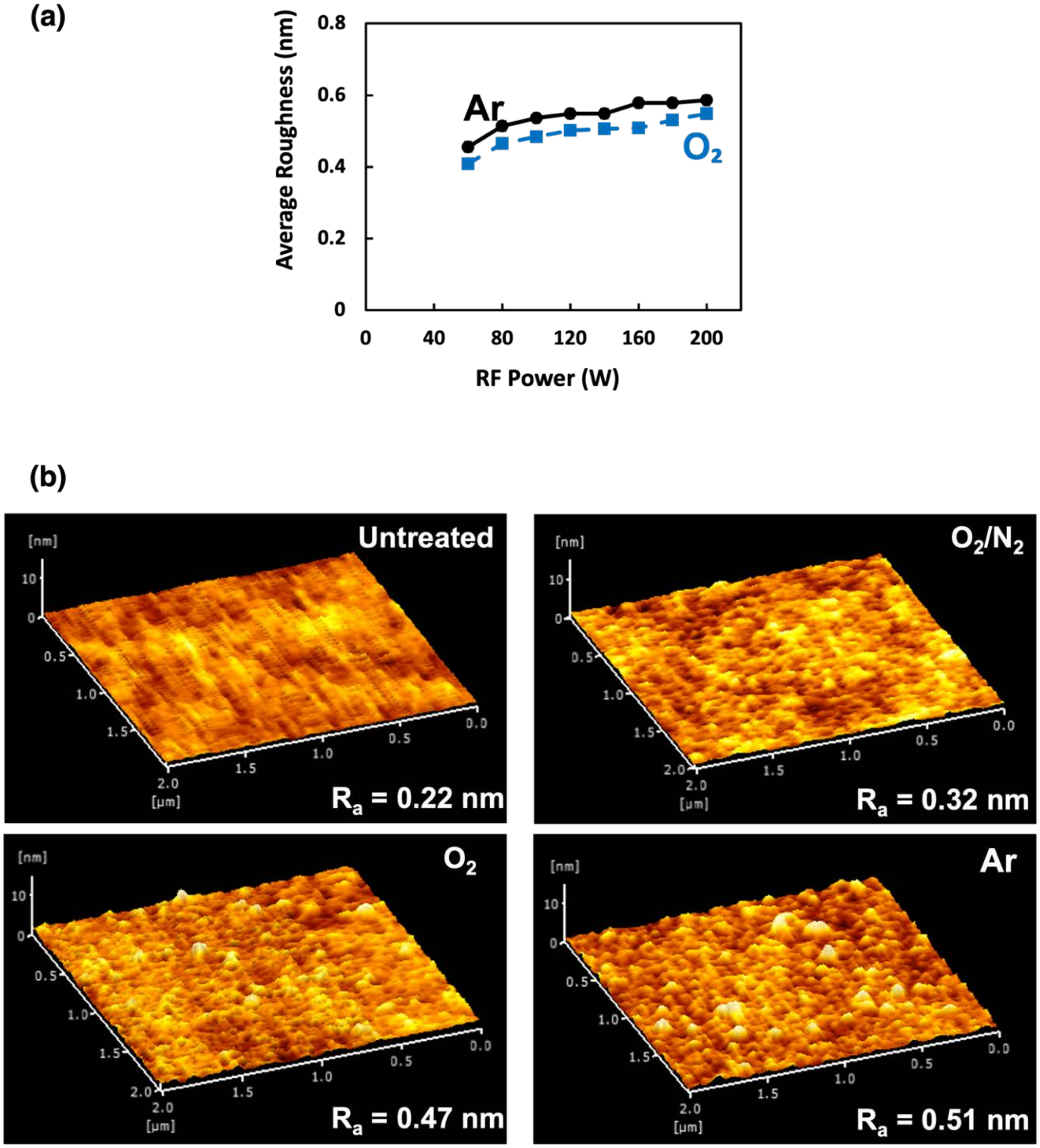

Supplementary Figure S1. Average roughness of plasma treated polydimethylsiloxane (PDMS). (**a**) Average roughness of O_2- and Ar-treated PDMS surfaces with RF power ranging from 60 to 200 W. (**b**) Three-dimensional images of surface roughness measured by atomic force microscope for PDMS surfaces that were untreated, oxygen/nitrogen (O_2/N_2) plasma at 200 W, and O_2 and Argon (Ar) plasma at 80 W.

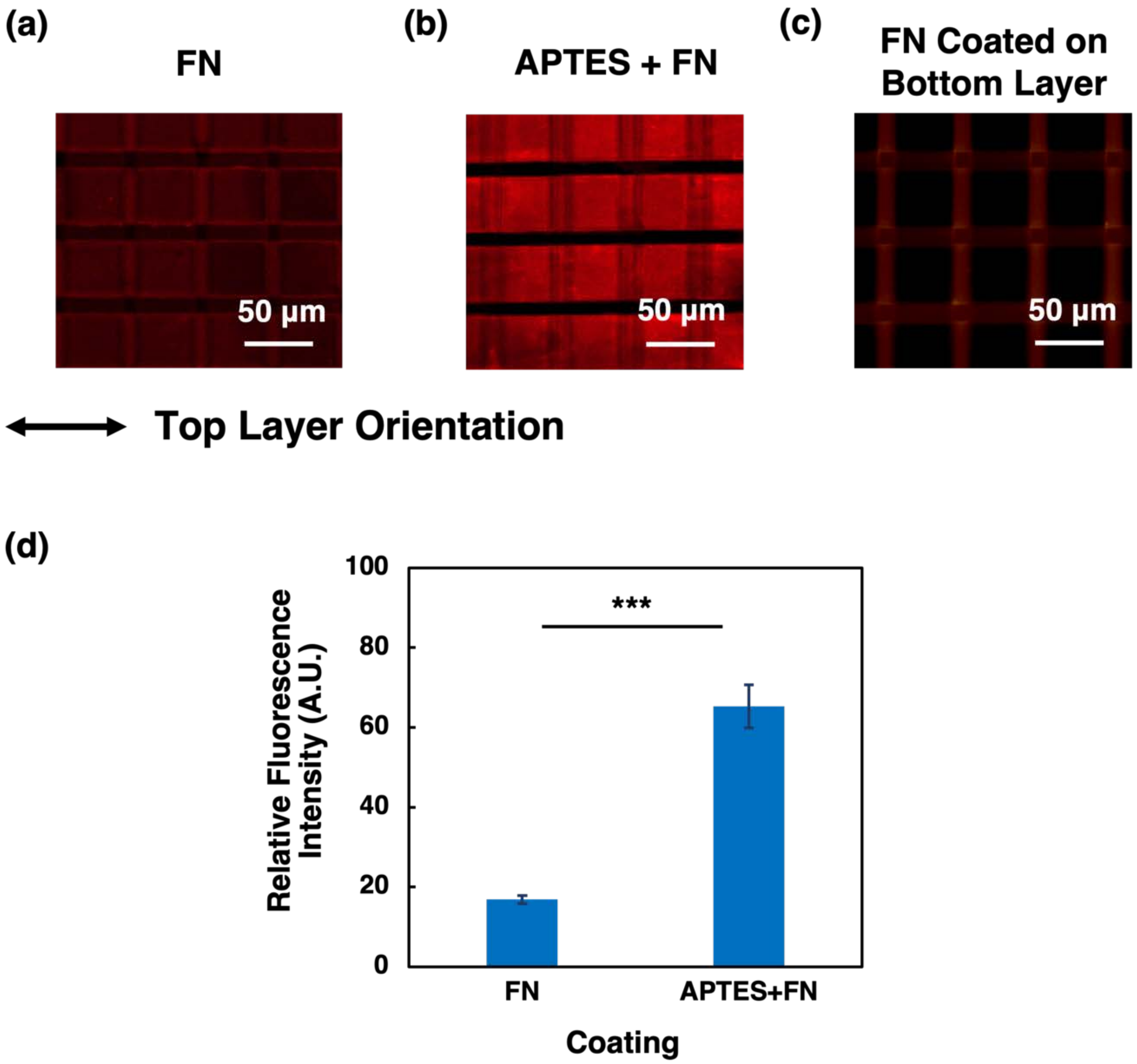

Supplementary Figure S2. Fluorescent imaging of FN. Fluorescence signals of (**a**) FN and (b) APTES ((3-aminopropyl)triethoxysilane) + FN coated on entire platforms, and (**c**) FN coated only on top layer sidewalls and bottom layer. (**d**) Relative fluorescence intensity of FN- and APTES + FN-coated platforms. The fluorescence intensity is relative to the background intensity. One-way ANOVA with Tukey's post hoc test; *** $p < 0.001$.

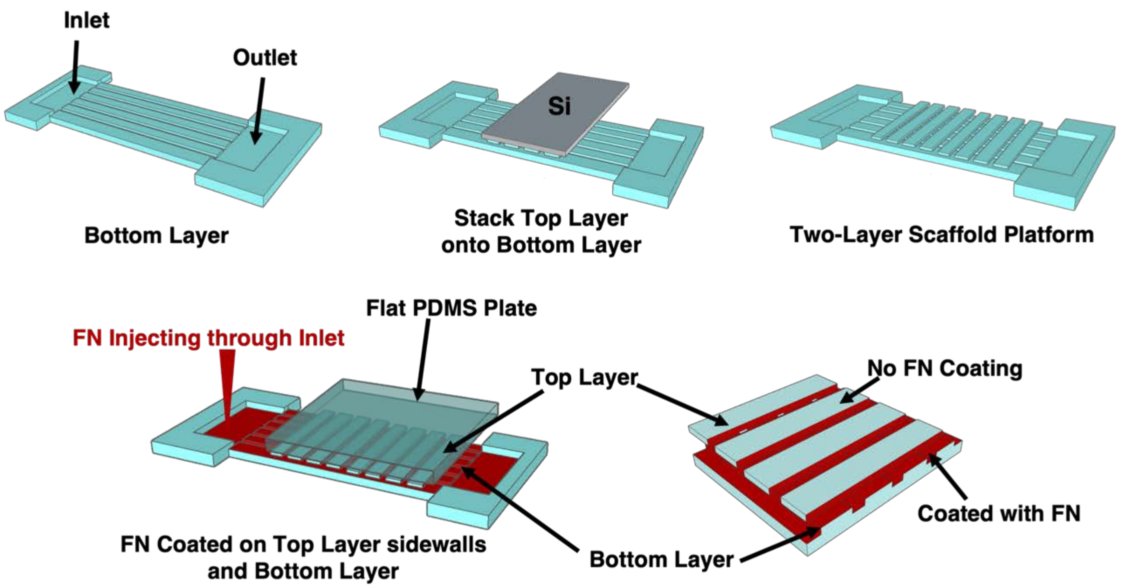

Supplementary Figure S3. Fabrication technology of coating fibronectin (FN) on top layer sidewalls and bottom layer of two-layer scaffold platform.
